# Dysregulation of Innate and Adaptive Immune Responses in Asymptomatic SARS-CoV-2 Infection with Delayed Viral Clearance

**DOI:** 10.7150/ijbs.72963

**Published:** 2022-07-11

**Authors:** Shanshan Cao, Qing Zhang, Liu Song, Mingzhong Xiao, Yexing Chen, Dianbing Wang, Min Li, Jinchao Hu, Lin Lin, Ying Zheng, Kun Zhou, Shujian Ye, Juan Zhou, Ya Na Zhou, Jing Cui, Jingzhi Wang, Jing Sun, Junxiu Tao, Zhou Chen, Rong Chen, Peng Zhou, Zhengli Shi, Sheng Wei, Linhua Zhao, Hui Wang, Xiaoling Tong, Xiaodong Li, Dong Men, Baidong Hou, Xian-En Zhang

**Affiliations:** 1 National Laboratory of Biomacromolecules, Institute of Biophysics, Chinese Academy of Sciences, Beijing 100101, China; 2 State Key Laboratory of Virology, Wuhan Institute of Virology, Center for Biosafety Mega-Science, Chinese Academy of Sciences, Wuhan 430071, China; 3 Hubei Provincial Hospital of Traditional Chinese Medicine, Wuhan 430074, China; 4 Guang'an Men Hospital, China Academy of Chinese Medical Sciences, Beijing 100053, China; 5 Wuchang District Health Bureau, Wuhan 430060, China; 6 Department of Epidemiology and Biostatistics, School of Public Health, Tongji Medical College, Huazhong University of Science and Technology, Wuhan 430030, China; 7 Faculty of Synthetic Biology, Shenzhen Institute of Advanced Technology, Chinese Academy of Sciences, Shenzhen 518055, China; 8 Hubei University, Wuhan 430074, China; 9 Wuchang District Hospital of Traditional Chinese Medicine, Wuhan 430060, China; 10 Jiyuqiao Street Community Health Service Center, Wuchang District, Wuhan 430081, China; 11 University of Chinese Academy of Sciences, Beijing 100049, China

**Keywords:** COVID-19, SARS-CoV-2, asymptomatic infection, inflammatory cytokine, immune repertoire, epidemiological investigation

## Abstract

Asymptomatic infection with SARS-CoV-2 is a major concern in the control of the COVID-19 pandemic. Many questions concerning asymptomatic infection remain to be answered, for example, what are the differences in infectivity and the immune response between asymptomatic and symptomatic infections? In this study, based on a cohort established by the Wuchang District Health Bureau of Wuhan in the early stage of the COVID-19 pandemic in Wuhan in 2019, we conducted a comprehensive analysis of the clinical, virological, immunological, and epidemiological data of asymptomatic infections. The major findings of this study included: 1) the asymptomatic cohort enrolled this study exhibited low-grade but recurrent activity of viral replication; 2) despite a lack of overt clinical symptoms, asymptomatic infections exhibited ongoing innate and adaptive immune responses; 3) however, the immune response from asymptomatic infections was not activated adequately, which may lead to delayed viral clearance. Given the fragile equilibrium between viral infection and host immunity, and the delayed viral clearance in asymptomatic individuals, close viral monitoring should be scheduled, and therapeutic intervention may be needed.

## Introduction

The coronavirus disease 2019 (COVID-19) caused by SARS-CoV-2 has resulted in a pandemic and a global epidemiological crisis since 2020. Unprecedented large-scale PCR screening and routine PCR testing in high-risk regions has identified large numbers of patients that had positive viral nucleic acid tests but were found to exhibit no concomitant clinical signs nor typical COVID-19 computed tomography (CT) images in their full course of infection 1, which made these cases distinct from symptomatic infections 2, 3. The occurrence of asymptomatic infections in patients who had tested positive for SARS-CoV-2 has varied from study to study 4-10. For example, an early study of the SARS-CoV-2 outbreak on the Diamond Princess cruise ship in Japan indicated that 17.9% of the 634 infected passengers were asymptomatic 11. Another study examined the results from 16 cohorts between 19 April and 26 May 2020 and found that the prevalence of asymptomatic infections varied in each cohort, with asymptomatic infections accounting for 40% to 45% of the population 12. Furthermore, with the appearance of new SARS-CoV-2 mutants, new evidence has suggested that the prevalence of asymptomatic infection is increasing 13, 14, which makes the threat of asymptomatic infection toward public health more complex.

Despite great clinical and research efforts, our understanding of the causes of asymptomatic SARS-CoV-2 infections is still very limited. While it is generally appreciated that the clinical symptoms of COVID-19 are the manifestations of inflammation caused by the host immune system in response to viral replication and cellular damage, it is surprising that no overt inflammatory responses are occurring in asymptomatic patients when there is clear evidence of viral replication in the upper respiratory tract of these patients. Recently, several studies have provided evidence of the activation of a response from both the innate and the adaptive arms of the host immune system in asymptomatic infection 15-17. Some studies have even suggested that efficient adaptive immunity was readily established in asymptomatically infected patients. However, other studies have suggested that the immune responses were suboptimal in asymptomatic infections compared with symptomatic COVID-19 infections 18. Thus, more research is needed to understand the underlying immune mechanisms of asymptomatic SARS-CoV-2 infection.

In this study, we investigated SARS-CoV-2 asymptomatic infection in a cohort established by the Wuchang District Health Bureau of Wuhan. We performed a comprehensive, integrated examination of asymptomatic infections using clinical observations, virology, immunology, and epidemiology. We detected low-grade but recurrent activity of viral replication in patients with asymptomatic infections and confirmed that asymptomatic infection was accompanied by ongoing innate and adaptive immune responses. In addition, we provided evidence that asymptomatic infections did not result in an adequate adaptive immune response, which may lead to delayed viral clearance.

## Materials and Methods

### Characteristics of the asymptomatic infection cohort

The study cohort enrolled 41 individuals with asymptomatic infections. Subjects were identified as being positive for SARS-CoV-2 by opportunistic screening after confirmed SARS-CoV-2-infected patients "zeroed out" in the Wuhan outbreak, including 36 cases from city-wide SARS-CoV-2 screening, 4 cases from community screening, and 1 case from hospital physical examination. Asymptomatic infections were classified as being confirmed positive in a viral nucleic acid test with no concomitant clinical signs nor typical COVID-19 CT image results according to the seventh edition of the Protocol for Prevention and Control of COVID-19 (PPCC). None of the cohort members had any self-perceived or clinically recognizable signs or symptoms and all the cohort members fulfilled the criteria of the PPCC.

In the cohort, there were 18 males (43.9%) and 23 females (56.1%), and the average age was 53.1 years. The comorbidities in the cohort included nine individuals (22.0%) with high blood pressure, five (12.2%) with diabetes, two (4.9%) with viral hepatitis, and one (2.4%) with thyroid disease. After enrollment, the vital signs (respiration, heart rate, blood pressure, pulse, and temperature), routine hemoanalysis and blood biochemical analysis, and routine urinalysis were regularly checked or examined. No clinical signs or test results of any infection were detected during the study period.

### Epidemiological investigation of the risk of viral transmission

For enrolled subjects, information on asymptomatic infections in close contacts was matched with information on COVID-19 infections reported by the Wuchang Sanitation and Health Bureau and the Chinese Center for Disease Control and Prevention (CDC) infectious disease report information management system to determine the status of infections among close contacts. After the matching was completed, the personal information was concealed, and the data were analyzed to assess the infectiousness of people with asymptomatic infections. Follow-up investigations were continued after patients had been discharged from the hospital.

### RT-PCR testing for SARS-CoV-2

Nucleic acid testing was performed on pharyngeal swabs and saliva samples from the patients. The samples were homogenized and the RNA was extracted using TRIzol. The DAAN Gene 2019-nCoV nucleic acid test kit (fluorescent PCR method) was used in the virological and immunological research. All operations were performed according to the manufacturers' instructions and samples with an *ORF1ab* cycle threshold (Ct) value <50 were considered positive.

### Serological testing for SARS-CoV-2 specific antibodies

Serum IgG and IgM were measured using a chemiluminescence immunoassay kit from Shenzhen Mindray Bio-Medical Electronics Co, Ltd, Shenzhen, China. N protein and spike protein receptor-binding domain (S-RBD) -conjugated magnetic particles were used to capture antibodies in serum samples. Then, alkaline phosphatase-conjugated anti-human COVID-19 specific IgG and IgM were added to the captured magnetic particles to form dual-antibody sandwich complexes. Finally, 3-(2'-spiroadamantyl)-4-methoxy-4-(3''-phosphoryloxy)-phenyl-1,2-dioxetane substrate was added and the luminescence intensity at 540 nm was detected. The unit of measurement for IgG was AU/mL, and the cutoff value was 10. The unit of measurement for IgM was COI, and the cutoff value was 1 (COI ≥1, positive and COI <1, negative).

### Detection of SARS-CoV-2 N protein

The SARS-CoV-2 N protein assay was performed using a fluorescence immunoassay lateral flow quantitative method provided by Beijing Savant Biotechnology Co., Ltd. The detection system contained diluted solution, captured N protein antibody (mouse antibody 49132), detection antibody (mouse anti-body 49132)-labeled latex fluorescent microspheres, a nitrocellulose membrane test strip, and a Savant-100 fluorescence immunoassay chromatography analyzer. The labeled IgG antibody against N protein and labeled rabbit IgG antibody were mixed and sprayed onto a glass fiber membrane to make a marker pad. The captured antibody was coated onto the nitrocellulose membrane as the test line (T line). The goat-anti-rabbit secondary antibody was coated onto the nitrocellulose membrane to make a quality-control line (C line). The marker pad was covered at the end of the nitrocellulose membrane close to the test line, and a water absorbent pad was covered at the other end of the nitrocellulose membrane close to the C line. The resultant fluorescence immunoassay lateral flow test strip for SARS-CoV-2 nucleocapsid protein was combined with a Savant-100 fluorescence immunoassay chromatography analyzer to produce a quantitative detection system.

Pharyngeal swabs and saliva samples of the cohort subjects were collected and homogenized. Thirty microliters of a sample were mixed with 30 μL of sample dilution buffer and incubated for 5 min. The mixture was dropped into the sample hole of the test trip, which was then left horizontal for 15 min to confirm that the C line of the test trip was continuous and clearly visible to the naked eye. Finally, the test strip was placed into the detector to read the fluorescence signal. According to the results of preliminary tests, a read signal <0.025 was defined as negative, and a read signal ≥0.025 was defined as positive (T/C ≥0.025).

### Analysis of BCR and TCR immune repertoire in peripheral blood mononuclear cells

To examine changes in the B cell receptor (BCR) and T cell (TCR) receptor composition in infected patients, peripheral blood mononuclear cells were separated from 10 mL of freshly collected peripheral blood. RNA was extracted from 8 × 10^6^ peripheral blood mononuclear cells using 1 mL of TRIzol. To generate libraries for immune repertoire analysis, the RNA samples were reverse transcribed and amplified using a switch-template PCR protocol to obtain DNA sequences for BCR IgM, IgG, and IgA heavy chains and IgK and IgL light chains, and for the TCR, each with custom designed primers. The libraries were then obtained by nested PCR with the addition of DNA barcodes. Pair-end (250 bp × 2) sequencing was used to obtain BCR/TCR sequences, and WIRED software (custom designed using the international ImMunoGeneTics database methodology as described in separate reports) was used for sequence assembly and further analysis. To quantify the diversity of an immune repertoire, the Hill index was used according to a previous report 19. In brief, Hill numbers are a class of diversity measures widely used in ecological studies as they integrate species richness and abundance. They are defined as



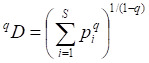



where *S* is the number of species in the assemblage and* p_i_* is the relative abundance*.* The parameter *q* determines the sensitivity of the measure to the relative frequencies. When *q* = 0, the relative frequencies contribute the least and the formula yields the sample species richness (number of species present). Using the Hill number formula, as well giving sample richness, also permits interpolation or extrapolation of the data, making it possible to compare diversity indexes between samples at the same data points despite sampling variances.

We used iNext 20, an R package, to calculate Hill numbers and compared the results at the data point of 20,000 samples. The number 20,000 is chosen because it is the number of sequences typically obtainable using the procedures described above, so the extent for data extrapolation is minimized.

### Detection of inflammatory cytokines

Inflammatory cytokines were detected using a cytometric bead array kit from Shenzhen Uni-Medica Science and Technology Co., Ltd. The microspheres with a given volume size and fluorescence intensity [652/622 nm signals of allophycocyanin (APC)] were coated with specific capture antibodies for each cytokine to form cytokine capture microspheres. The fluorescence intensity was used to measure 12 cytokines, including interleukin (IL)2, IL4, IL6, IL10, tumor necrosis factor (TNF), and interferon-γ. The microspheres were co-incubated with a standard or the samples and biotin-labeled detection antibodies to create a double antibody sandwich complex. After the reaction of this sandwich complex with streptavidin-modified phycoerythrin (SA-PE), the signals of APC and PE were detected using a flow cytometer, where the intensity of the APC signal reflected the type of cytokine in the sample, and the intensity of the PE fluorescence reflected the quantity of the corresponding cytokine.

According to the operation manual, the specific experimental steps were: 1) centrifuge the patient's plasma sample at 1,000 × g for 10 min at 4°C and remove the supernatant; 2) mix 25 µL of plasma supernatant with 25 µL of vortexed capture microspheres and 25 µL of detection antibody, incubate for 2 h at room temperature with protection from light; 3) add 25 µL of fluorescent detection reagent (SA-PE) to each tube, continue to incubate for 0.5 h at room temperature with protection from light; 4) add 500 µL of washing buffer to each tube, vortex for a few seconds, centrifuge at 4°C and 500 × g for 5 min, carefully pour off the supernatant and remove the excess liquid from the mouth of the tube by touching on absorbent paper; 5) add 100-300 µL of washing buffer to each tube, vortex for 20-30 s and resuspend the microspheres before fluorescence detection. Data were obtained by flow cytometry (BD LSRFortessa) and analyzed using LEGENDplex v.8.0 (VigeneTech).

## Results

### The risk of viral transmission from the asymptomatic cohort was low

Of the 41 individuals enrolled in the cohort, 37 had a total of 107 close contacts. Eighty-two of these close contacts were exposed at home by sharing meals and living in the same room. One close contact was exposed at a medical institution, and the mode of exposure was consultation and care. The remaining 18 close contacts were exposed in the workplace by working and studying in the same room. All close contacts had no signs or symptoms related to COVID-19 and tested negative for SARS-CoV-2 nucleic acid.

### Asymptomatic infection exhibited low-grade viral replication

**Figure 1:**
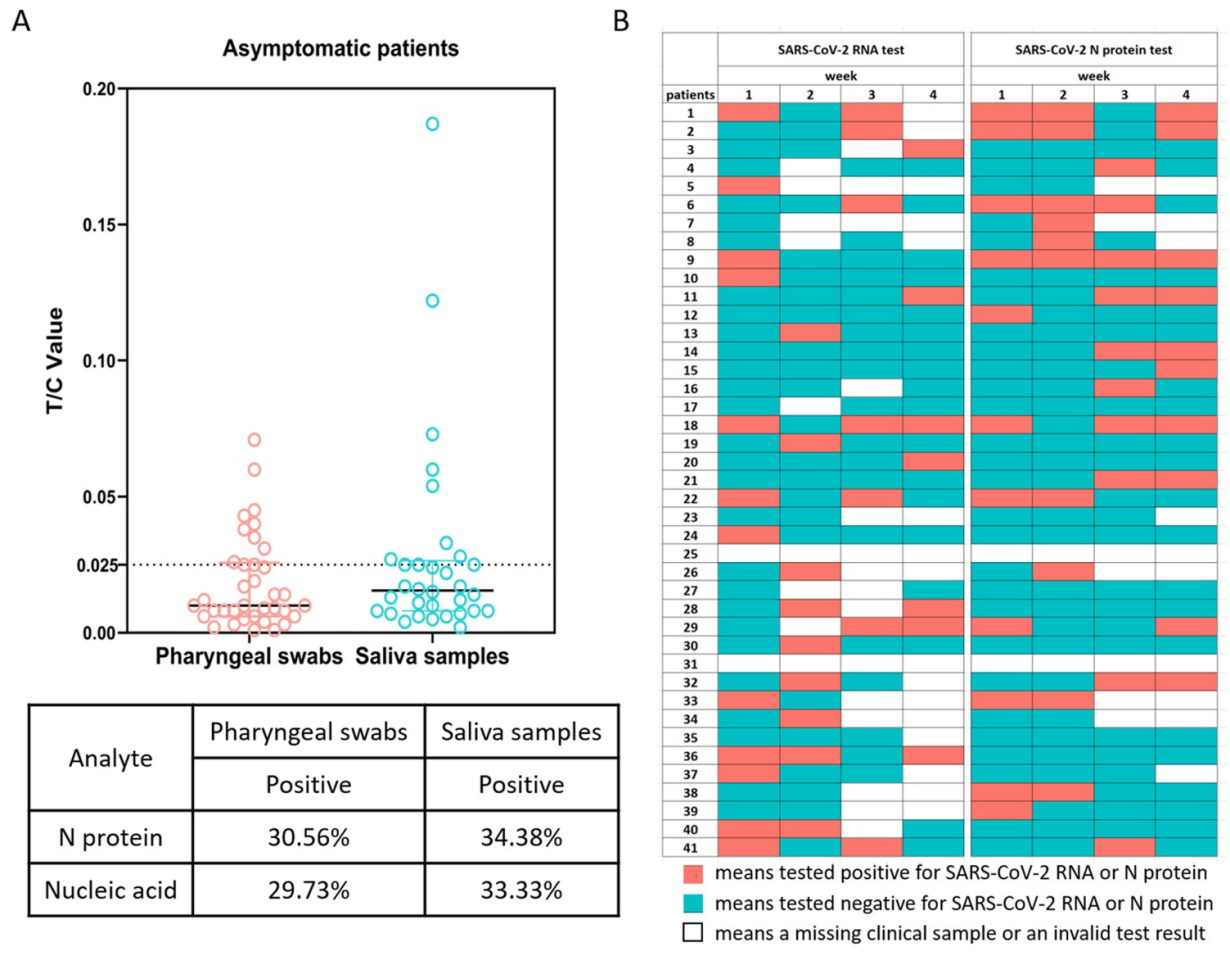
SARS-CoV-2 RNA and N protein in pharyngeal swabs and saliva samples. (A) SARS-CoV-2 N protein in pharyngeal swabs and saliva samples from individuals with asymptomatic infections at 1 week were measured by a fluorescence-based lateral flow immunoassay. The concentration of N protein is presented as the ratio of the fluorescent intensity of the T and C lines with a cutoff value of 0.025. The table shows the statistical analysis and comparison of SARS-CoV-2 N protein in pharyngeal swabs and saliva samples. (B) Recurrent positive tests for SARS-CoV-2 RNA and N protein in individuals with asymptomatic infections over 4 weeks.

The SARS-CoV-2 RNA tests of all the cohort members were positive on admission to hospital. One week after enrollment, the number of positive results for the viral nucleic acid tests of the pharyngeal swabs and saliva samples decreased greatly. Testing for the viral N protein in the pharyngeal swabs and saliva samples also confirmed the low positive rate of virus detection (Figure 1A). We noted that there was some discrepancy in the positive tests between these two methods. Because the Ct values of the viral nucleic acid tests were generally quite high, and the concentrations of the N protein in most of the tests were close to the detection limit, we reasoned that this discrepancy was likely because of the low viral RNA and antigen loads in the samples from the upper respiratory tract of asymptomatically infected individuals. In support, for eight individuals, we failed to isolate live virus from their sputum samples collected on the day of enrollment. Interestingly, for many individuals that tested negative at week 1, subsequent weekly tests could become positive again for SARS-CoV-2 RNA or N protein (Figure 1B). These results suggested that there might be continuing viral infection in this cohort with asymptomatic infections.

### IgG levels in asymptomatic infections were approximately half those in patients recovering from symptomatic infections

**Figure 2:**
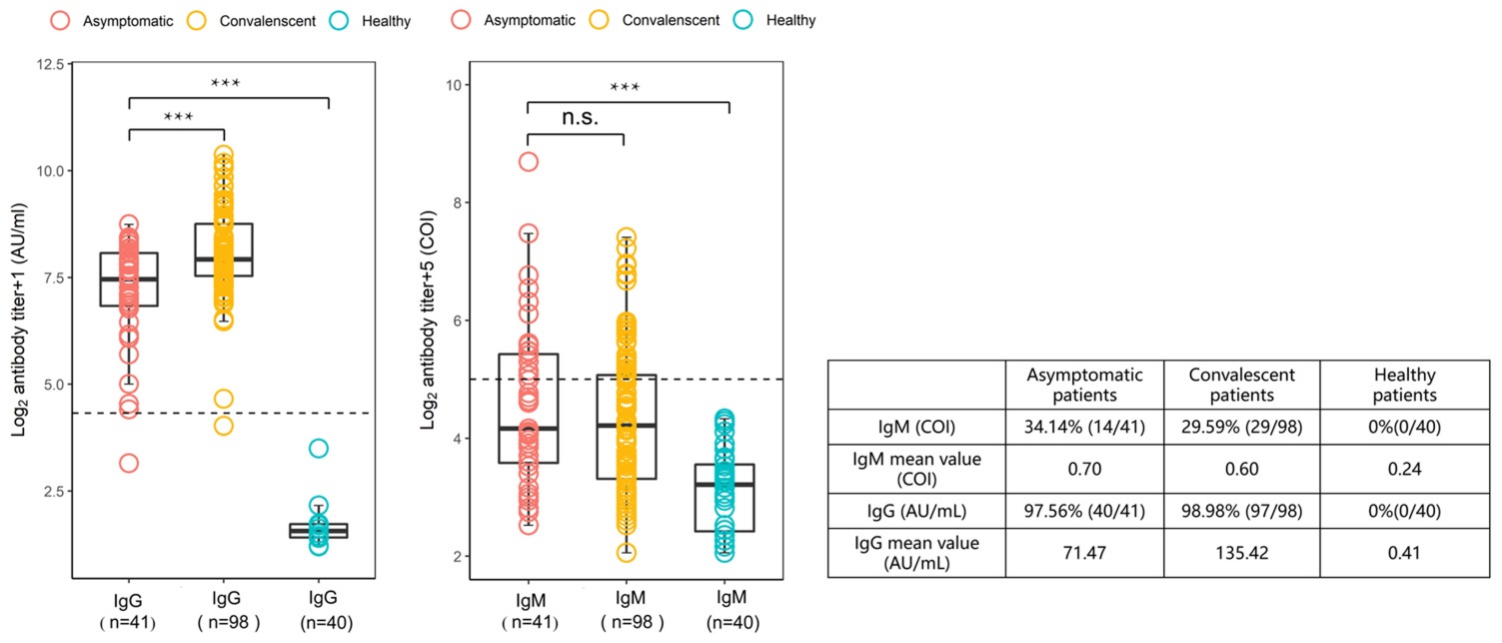
Comparison of the levels of SARS-CoV-2-specific IgG and IgM antibodies in asymptomatic infected individuals, COVID-19 recovering patients, and healthy controls. Plasma samples from the asymptomatic group (n = 41) were collected and measured during the 1^st^ week of the patients' hospitalization and samples from recovered patients (n = 98) were collected 19-23 weeks after discharge from the hospital. The IgG antibody levels were measured in AU/mL with a cut-off value of 10 AU/mL. The levels of IgM antibodies are presented as the measured relative light units divided by the cutoff value (cutoff index, COI): COI ≥1 was defined as positive and COI <1 as negative. The box plot shows the median (middle line) and the first quartile and third quartile (boxes), and the 1.5 times quartile spacing is shown above and below the boxes. The table shows the statistical analysis and comparison of the two sets of sample measurements.

To characterize the SARS-CoV-2-specific antibody response in individuals with asymptomatic infections, plasma samples from asymptomatic infected patients within week 0/1 (2-3 days) of hospital admission were analyzed. The results are shown in Figure 2, compared with the plasma samples from 98 patients with symptomatic infections at weeks 19-23 of recovery. In individuals with asymptomatic infections, 34.14% (14/41) were positive for IgM antibodies and 97.56% (40/41) were positive for IgG antibodies, and in recovering patients, 29.59% (29/98) were IgM positive and 98.98% (97/98) were IgG positive. Among the asymptomatic infections, the geometric mean of IgM (median 0.56, 0.35-1.36) was 0.7 COI and the geometric mean of IgG (median 87.98, 55.88-135.55) was 71.47 AU/mL; while the geometric mean of IgM (median 0.57, 0.31-1.06) in recovering patients was 0.6 COI and the geometric mean of IgG (median 121.60, 92.61-218.14) was 135.42 AU/mL. From the above results, the IgG levels in individuals with asymptomatic infections were found to be approximately half those in patients recovering from symptomatic infections, suggesting that the anti-viral IgG antibody response was attenuated in these asymptomatic individuals.

### Asymptomatic infection did not exhibit an active adaptive immune response in peripheral lymphocyte populations

**Figure 3:**
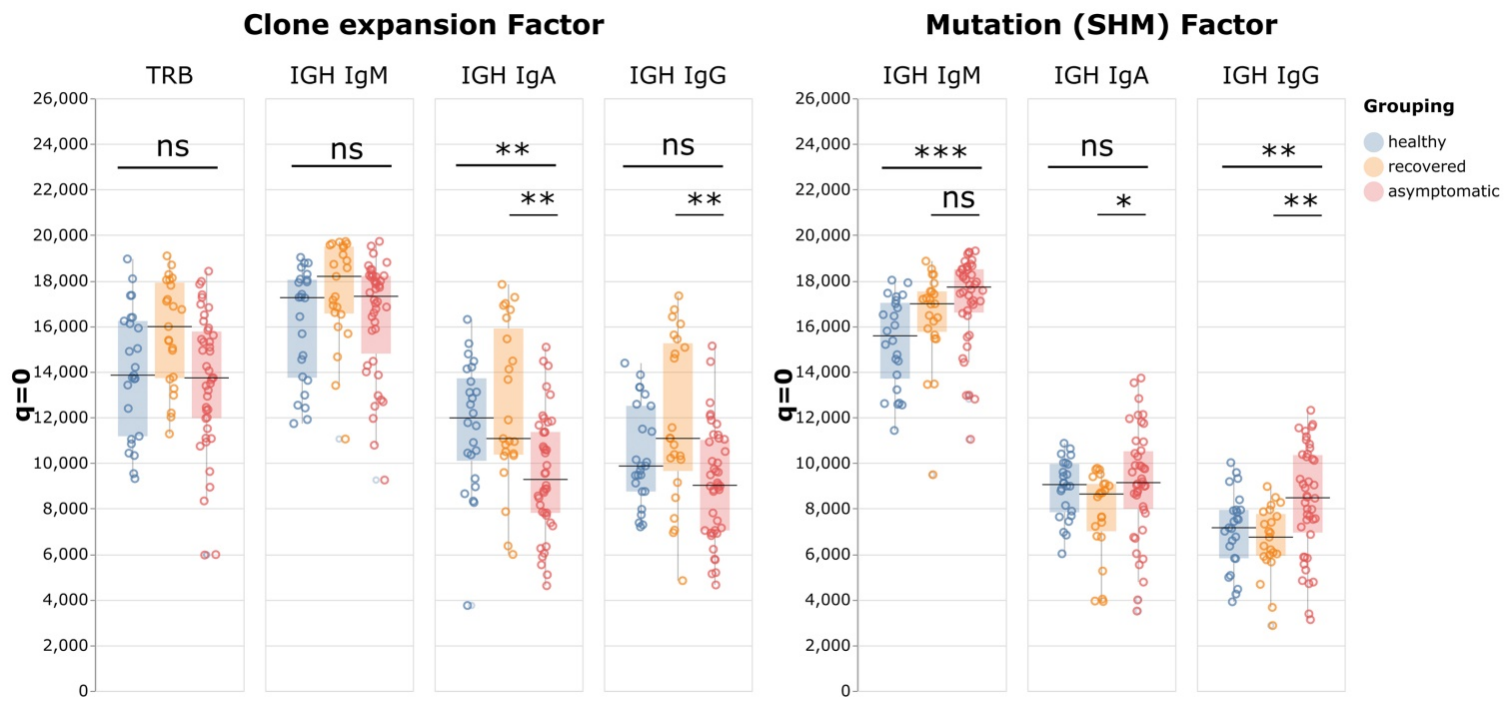
Peripheral blood immune repertoire diversity indexes (Hill numbers) for asymptomatic individuals with positive COVID-19 tests. In both charts *q* = 0 and *S* = 20,000, and each point corresponds to the “species richness” when the sample contained 20,000 data points for one individual. The left panel shows the numbers of sequence clones given 20,000 total sequences, which indicates the various degrees of clone expansion in the asymptomatic group (n = 41), the healthy group (n = 22), and the recovered group (n = 23). The right panel shows, for the same groups, the number of VDJ clones given 20,000 different sequence clones, which is indicative of the SHM factors.

A typical adaptive immune response to infection features clonal expansion of antigen-specific B and T cells, which results in reduction of repertoire diversity of sampled cells. To examine whether activation of adaptive immune cells was affected in patients with asymptomatic infections, the repertoire diversities of T cells and B cell subsets were compared using sequence clone (defined as a unique RNA sequence) information. When a clone expands, it is likely the same sequence would be seen multiple times (with different Unique Molecular Identifiers) in the same sample, so we used the “sizes” of sequence clones to calculate the Hill numbers reflecting clone expansion factors. A high number means that most sequences have one copy sampled, indicating little expansion. The further the number is below 20,000, the more active expansion it represents. Surprisingly, the Hill numbers of neither TCR beta nor IgM BCR sequences were appreciably different between asymptomatic patients and healthy control groups, suggesting that these cells were not undergoing clonal expansion, even in active viral infections (Figure 3). In the class-switched populations, the Hill numbers for IgG exhibited a decreasing trend in the asymptomatic group but this did not reach statistical significance, whereas those for the IgA repertoires were lower than in the other two groups, indicating there were more expanded clones in this B cell subset.

Somatic hypermutation (SHM) is a critical process in activated B cells to generate high affinity antibodies and contributes to BCR repertoire diversity. Therefore, by analyzing these mutations, we could identify relationships between clones with the same germline sequence, and gain insights into the antibody affinity maturation process in relation to antigen stimulation. To evaluate the effect of SHM on BCR repertoire diversity in asymptomatic infection, first the BCR clones with the same Complementary Determining Region 3 (CDR3) and V and J allele usage were identified as germline "VDJ clones", and a difference between any sequence clone and its VDJ clone was identified as a mutation caused by SHM. For example, if five sequence clones belonged to one VDJ clone, it is the equivalent of finding five individuals of one species (the VDJ clone) in a sample. These sequence clone vs VDJ clone ratios were then used as data points to calculate the Hill numbers. When *q* = 0 and *S* = 20,000, the resulting number indicates how many VDJ clones are expected to be identified among 20,000 unique sequence clones. If the number is close to 20,000, it means that most VDJ clones manifest as only one sequence clone, indicating the sequence diversity mostly came from the VDJ recombination process, with little contribution from SHM. The further the number is below 20,000, the more SHM contributes to BCR diversity. The results showed that for all immunoglobulin isotypes, the Hill numbers for the asymptomatic group were higher than those of the recovered patients. This result indicated that the asymptomatic group had less diversification from SHM than the recovered group, especially in IgA and IgG (p-values: IgM p = 0.06; IgA p = 0.01; and IgG p = 0.003).

### Asymptomatic infection was associated with dysregulation of inflammatory cytokine production

**Figure 4:**
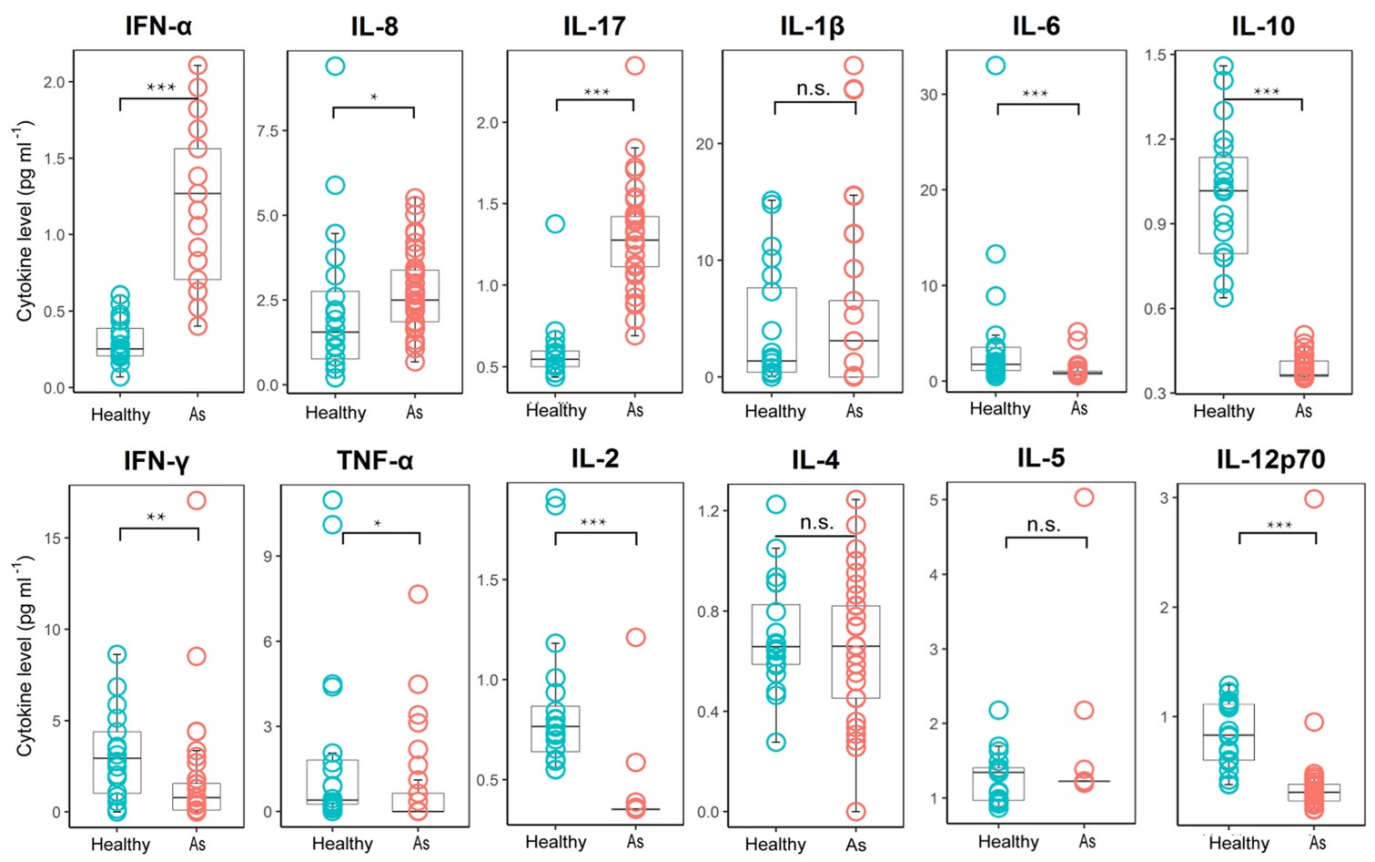
Comparison of the plasma cytokine levels between patients with asymptomatic infections and healthy subjects. Plasma samples from subjects with asymptomatic infections (n = 41) were collected in the acute stage in hospital and tested for cytokine concentrations, and healthy group (n = 20) cytokine concentrations were obtained from the plasma sample data of healthy people from normal physical examinations (provided by the Shenzhen Uni-Medica Science and Technology Co., Ltd.). The box plots display the median values (intermediate line) and the first quartile and the third quartile (box) and 1.5 times the interquartile range shown above and below the box. Unpaired, dual-sided Mann-Whitney U test p values are indicated: * p < 0.05, ** p < 0.01, and *** p < 0.001. As, asymptomatic.

To further investigate the immune response in patients with asymptomatic infections, we examined the serum cytokine levels. Type I interferons are produced by host cells when infected by a virus and have broad-spectrum anti-viral effects, such as the direct inhibition of viral replication. Interestingly, most of the asymptomatically infected patients exhibited increased serum levels of interferon-α (IFNα), compared with the healthy control group (Figure 4), indicating that the innate immune system of the host can detect the infecting virus in asymptomatic infections. In addition, most asymptomatic patients also exhibited increased levels of IL-17, an important anti-bacterial and anti-fungal cytokine, and IL-8, a chemokine for recruiting neutrophils. Elevated levels of IL-17 and IL-8 are often associated with bacterial infections or inflammatory diseases mediated by neutrophils but usually play little part in the anti-viral immune response. Two recent studies have reported that SARS-CoV-2 infection can induce S100A8/A9, an endogenous toll-like receptor 4 ligand, which caused a neutrophil-mediated immune disorder 21,22. Thus, induction of these cytokines and chemokines in asymptomatic infection might be an immune evasion mechanism of SARS-CoV-2. The serum levels of IL-1β, a potent pro-inflammation cytokine, were not increased in the asymptomatic group, consistent with the lack of overt clinical symptoms in these individuals. In addition, the levels of pro-inflammatory cytokines, such as TNFα and IL-6, in the asymptomatic group were lower than those in the healthy control group. Similarly, the levels of the T-helper 1 (Th1)-promoting lymphokine, IL-2, were also greatly reduced. These results, together with the reduced levels of Th1 effector cytokines, such as IFNγ, and the reduced levels of serum IL-12p70, a Th1-promoting cytokine produced mainly by dendritic cells, in asymptomatic patients, indicated that asymptomatic infections were associated with a defective Th1 response. In contrast, there were no significant differences in the levels of Th2-associated cytokines, such as IL-4 and IL-5, in both groups. Unexpectedly, the levels of IL-10, an inhibitory inflammatory cytokine, were also reduced in the asymptomatic group. In summary, these results suggested that asymptomatic infection might be related to dysregulation of inflammatory cytokine production.

## Discussion

The major points of our study regarding viral infections in asymptomatic patients were: 1) all the asymptomatic patients were identified by a positive test for SARS-CoV-2 RNA; 2) viral N protein was detected in the upper respiratory tract samples from a large proportion of the patients; 3) the majority of the patients tested positive for anti-viral IgG, and a few patients also tested positive for anti-viral IgM; 4) increased serum IFNα levels were detected in most of the patients; but 5) no live virus could be isolated and cultured from the upper respiratory tract samples; and 6) all the close contacts had no COVID-19-related signs or symptoms and were negative for SARS-CoV-2 nucleic acid. These results indicated that the asymptomatic patients were in a process of active viral infection, but the activity of viral replication in the upper respiratory tract was weak.

The prolonged active viral infection indicated that the immune responses in the asymptomatic patients were distinct from those with regular SARS-CoV-2 infection. The SARS-CoV-2 specific IgG level in the asymptomatic group was substantially reduced compared with that of the recovered patients, indicating that the majority of asymptomatic infections induced a low level adaptive immune response. The results of immune repertoire analysis appeared to support this notion. Neither alpha/beta T cells nor IgM B cells of the asymptomatic group showed clear evidence of clonal expansion. In addition, only the IgA subset in the Ig-switched B cell populations of the asymptomatic group exhibited statistically significant signs of clonal expansion, whereas the IgG subset of B cells did not, which was consistent with the reduced SARS-CoV-2 IgG levels in these patients. Examination of SHM revealed there was a reduced effect of SHM on BCR diversity in the asymptomatic group, indicating that there might be defects in B and T cell interaction, e.g., in germinal centers. Defects in the germinal center reaction have been associated with poor outcomes in COVID-19 patients with severe infections 23, 24. In parallel, we found evidence from the serum cytokine data indicating that there was reduced activation and activity of the Th1 response. In anti-viral responses, CD4^+^ T cells are polarized to both Th1 and T follicular helper (Tfh) cells, with the former able to secrete cytokines, such as IFNγ and TNFα, and the latter able to assist B cells. Thus, defective CD4^+^ T cell activation and polarization can lead to reduced production of anti-viral cytokines and a poor antibody response. Because of the critical function of the adaptive immune system in sterilizing immunity, our data indicated that the failure to adequately activate adaptive immunity is likely to be the cause of defective viral clearance in asymptomatic patients.

The cause of the inadequate adaptive immune response in asymptomatic infection is not completely clear. The increased levels of serum IFNα in most of the asymptomatic patients means that it is unlikely that the virus can completely evade detection by the host's innate immune system. However, the failure to induce additional signals, such as Th1-promoting cytokine IL-12p70 or Tfh-promoting cytokine IL6, points to a lack of proper coordination between the two important arms of the host immune system. This lack of coordination could be caused by an immune evasion mechanism of the virus, i.e., diverting anti-viral immunity to an anti-bacterial response by promoting IL-17 production, thereby impairing the efficiency of the anti-viral response. Or, the lack of coordination could be caused by genetic factors that affect the interactions and coordination between immune cells. In the future, we will track lymphocyte activation while patients are undergoing treatment and in the process of clearing the virus. The results of this proposed study might shed light on the mechanism of delayed viral clearance in asymptomatic individuals.

## Conclusions

From the findings of this study of an asymptomatic infection cohort established in the early COVID-19 outbreak in Wuhan we drew the following conclusions:

1) The cohort with asymptomatic infection exhibited active but low-grade viral replication, and the risk of viral transmission from these individuals was low but cannot be excluded.

2) Both innate and adaptive immunity were activated in asymptomatic infection but clearly showed dysregulation and provided only partial immune protection, which may lead to delayed viral clearance.

From these conclusions, we strongly recommend that asymptomatic infections should be closely monitored for the following reasons.

First, although asymptomatic infections were shown not to be highly infectious in this study, the risk of viral transmission cannot be excluded given the fragile balance between viral infection and host immunity, which might lead to increased viral infectivity under some circumstances, such as immune suppression. Second, the cohort members in this study were screened 2 months after the peak of the epidemic in Wuhan; therefore, they might be at the end stage of infection, which does not preclude them from having been more infectious at an earlier stage. Third, the asymptomatic infections of this cohort were caused by early epidemic SARS-CoV-2 strains and, since then, a variety of variant epidemic strains with different characteristics have appeared. Therefore, the results of this study may not represent the asymptomatic infection characteristics caused by other variant epidemic strains.
